# Neoadjuvant immunochemotherapy in locally advanced esophageal squamous cell carcinoma: a retrospective study with 2-year survival analysis

**DOI:** 10.1007/s00432-025-06263-1

**Published:** 2025-07-17

**Authors:** Chong-Rui Li, Yu-Zhen Chen, Bin Li, Mei-Yu Ren, Yu-Qi Meng, Tie-Niu Song, Jian-Bao Yang, Cheng Wang, Xiao-Ping Wei, Peng Jiang, Duo-Jie Zhu

**Affiliations:** Department of Thoracic Surgery, The Second Hospital & Clinical Medical School, Lanzhou University, 82 Cuiyingmen, Chengguan District, Lanzhou, 730030 Gansu Province China

**Keywords:** Neoadjuvant immunochemotherapy, Esophageal squamous cell cancer, Disease-free survival, Overall survival, Pathological complete response

## Abstract

**Background:**

Neoadjuvant immunochemotherapy (NICT) has shown encouraging short-term outcomes in patients with locally advanced resectable esophageal squamous cell carcinoma (ESCC), but data on long-term survival remain limited. This study compared the therapeutic efficacy, safety, and 2-year survival outcomes of NICT versus surgery alone.

**Methods:**

We retrospectively analyzed patients with locally advanced resectable ESCC who underwent either NICT followed by surgery or upfront surgery alone. Primary endpoints were disease-free survival (DFS) and overall survival (OS).

**Results:**

A total of 188 patients were included, with 60 receiving NICT and 128 undergoing surgery alone. The NICT group achieved better 2-year DFS (76.7% vs. 57.0%, *P* = 0.021) and OS (86.7% vs. 68.0%, *P* = 0.0053), with similar rates of postoperative complications (40.0% vs. 37.5%, *P* = 0.742). No ≥ grade 3 postoperative complications occurred in the NICT group, while three cases (2.3%) were observed in the surgery-alone group. Pathological responses to NICT included 28.3% complete response (pCR) and 61.7% major response (MPR). Grade 3 treatment-related adverse events occurred in 20.0% of NICT patients, with no grade ≥ 4 events. Patients achieving pCR or MPR had significantly better survival outcomes than non-responders. Survival outcomes were similar between 2-cycle and > 2-cycle NICT regimens. ECOG performance status, coronary artery disease, and treatment modality were identified as independent prognostic factors.

**Conclusion:**

NICT followed by surgery demonstrated favorable pathological response and 2-year survival outcomes in locally advanced ESCC, supporting its potential as a neoadjuvant strategy pending further prospective validation.

## Introduction

Esophageal carcinoma (EC), one of the most prevalent epithelial malignancies, accounted for approximately 511,000 new cases and 445,000 deaths globally in 2022, ranking 11th in incidence and 7th in mortality among all cancers (Bray et al. 2024). Esophageal squamous cell carcinoma (ESCC) constitutes the predominant histological subtype, representing 90% of all EC cases (Jiang et al. 2025). For locally advanced resectable ESCC, multidisciplinary treatment involving neoadjuvant therapy followed by surgery remains a cornerstone strategy (Deboever et al. 2024; Zeng et al. 2024). While neoadjuvant chemotherapy (NCT) and neoadjuvant chemoradiotherapy (nCRT) have demonstrated short-term oncological benefits, concerns persist regarding their suboptimal safety profiles and limited long-term survival advantages (Leng et al. 2025; Liang et al. 2024; Cheng et al. 2024). This underscores the urgent need to explore novel neoadjuvant strategies for this patient population.

The advent of programmed cell death protein 1 (PD-1) inhibitors combined with chemotherapy has revolutionized systemic therapy, establishing this regimen as the first-line standard for advanced esophageal and gastroesophageal junction (GEJ) cancers (Li et al. 2022; Beshr et al. 2024). Building on these advancements, neoadjuvant immunochemotherapy (NICT) has emerged as a transformative approach, demonstrating remarkable potential in early-phase trials (Liu et al. 2023; Wang et al. 2024; Chen et al. 2024). Recent studies report that NICT achieves pathological complete response (pCR) rates of 21.7–50% with manageable toxicity profiles (Yang et al. 2024a; Chen et al. 2023; Guo et al. 2024; Wu et al. 2025). However, robust evidence evaluating the survival impact of NICT remains scarce. This study comprehensively assesses the efficacy, safety, and 2-year survival outcomes of NICT compared to surgery alone in locally advanced resectable ESCC.

### Study design and patient selection

We retrospectively reviewed consecutive patients with locally advanced ESCC who underwent either NICT followed by surgery or surgery alone at the Second Hospital of Lanzhou University between November 2019 and May 2023. The study protocol was approved by the Ethics Committee of the Second Hospital of Lanzhou University, with a waiver of informed consent due to the retrospective design. Anonymized data were extracted from our prospectively maintained institutional database. *Inclusion Criteria* (1) Age 18–75 years. (2) Histologically confirmed ESCC. (3) Clinical stage II–III disease per UICC/AJCC 8th edition TNM classification: cT1N2M0 or cT2-3N0-2M0, confirmed by thoracic/abdominal computed tomography (CT) and endoscopic ultrasound. (4) Completion of McKeown esophagectomy. *Exclusion criteria* (1) Prior neoadjuvant chemotherapy or chemoradiotherapy. (2) Concurrent malignancies or history of other cancers. (3) Previous antitumor therapies (radiotherapy, immunotherapy, or targeted agents). (4) Incomplete medical records. All staging evaluations adhered to the UICC/AJCC 8th edition TNM staging system.

### Neoadjuvant treatment protocol

Patients in the NICT group received at least two cycles of NICT, consisting of a PD-1 inhibitor (administered intravenously at 200 mg for pembrolizumab, camrelizumab, sintilimab, or tislelizumab) combined with platinum-based chemotherapy. The chemotherapy regimen included cisplatin 75 mg/m^2^ or carboplatin (target area under the curve, AUC = 5 mg/mL/min), and paclitaxel 175 mg/m^2^, all delivered intravenously on Day 1 of each 21-day cycle. Following the completion of two treatment cycles, patients entered a 4–6-week treatment-free interval for therapeutic response evaluation. Restaging assessments fwere performed using contrast-enhanced thoracic/abdominal CT and endoscopic ultrasound. Patients deemed eligible for surgery subsequently underwent McKeown esophagectomy within 2 weeks after restaging.

### Surgical intervention

Patients in the NICT group underwent surgery following a 4- to 6-week post-treatment observation period after completing at least two therapy cycles. All enrolled patients received standardized thoracoscopic-laparoscopic minimally invasive McKeown esophagectomy with systematic two-field lymphadenectomy, performed by designated thoracic surgeons with > 5 years of esophagectomy experience.

### Follow-up protocol

Postoperative surveillance was conducted according to National Comprehensive Cancer Network (NCCN) guidelines. Patients underwent scheduled evaluations every 3 months during the first 2 years and every 6 months thereafter. Routine assessments included physical examinations, contrast-enhanced chest CT, and barium swallow studies. Additional diagnostic modalities—such as abdominal/pelvic ultrasonography, upper endoscopy with biopsy (when indicated), whole-body 18F-fluorodeoxyglucose positron emission tomography/computed tomography (18F-FDG PET/CT), and magnetic resonance imaging (MRI) of the brain or bones—were selectively performed based on clinical suspicion of recurrence or metastasis. This protocol ensured systematic monitoring of treatment efficacy and early detection of disease progression.

### Study endpoints

The primary endpoints were disease-free survival (DFS) and overall survival (OS). DFS was defined as the interval (in months) from surgery to the first documented recurrence, metastasis, or death from any cause, while OS represented the duration from surgery to death or last follow-up (censored at the final contact). Secondary endpoints included pCR rate, major pathological response (MPR) rate, pathological downstaging rate, and treatment-related adverse events. pCR was defined as the absence of viable tumor cells in both the primary tumor and resected lymph nodes. MPR was characterized by residual viable tumor cells occupying ≤ 10% of the primary tumor bed area. Pathological downstaging referred to a reduction in post-treatment pathological T stage (ypT) and/or N stage (ypN) compared to pretreatment clinical staging.

### Statistical analysis

All statistical analyses were performed using R (version 4.3.2) and IBM SPSS Statistics (version 27.0). Categorical variables are expressed as frequencies and percentages [n (%)], with intergroup comparisons conducted using chi-square (χ^2^) or Fisher’s exact tests as appropriate. Normally distributed continuous variables were presented as mean ± standard deviation (SD) and analyzed with independent samples t-tests; non-normally distributed variables were reported as median with interquartile range (IQR) and assessed via Mann–Whitney U tests. The association between pathological response and radiological evaluation, along with the correlation between pathological outcomes and NICT cycle numbers, were evaluated using χ^2^ or Fisher’s exact tests. Survival analyses included: estimation of median follow-up duration via the reverse Kaplan–Meier method, generation of Kaplan–Meier curves for DFS and OS, and comparison of intergroup survival outcomes through log-rank tests; univariate and multivariate Cox regression analyses were performed to identify independent prognostic factors for DFS and OS. All tests were two-sided, with statistical significance defined at *P* < 0.05.

## Results

### Patient characteristics

**Fig. 1:**
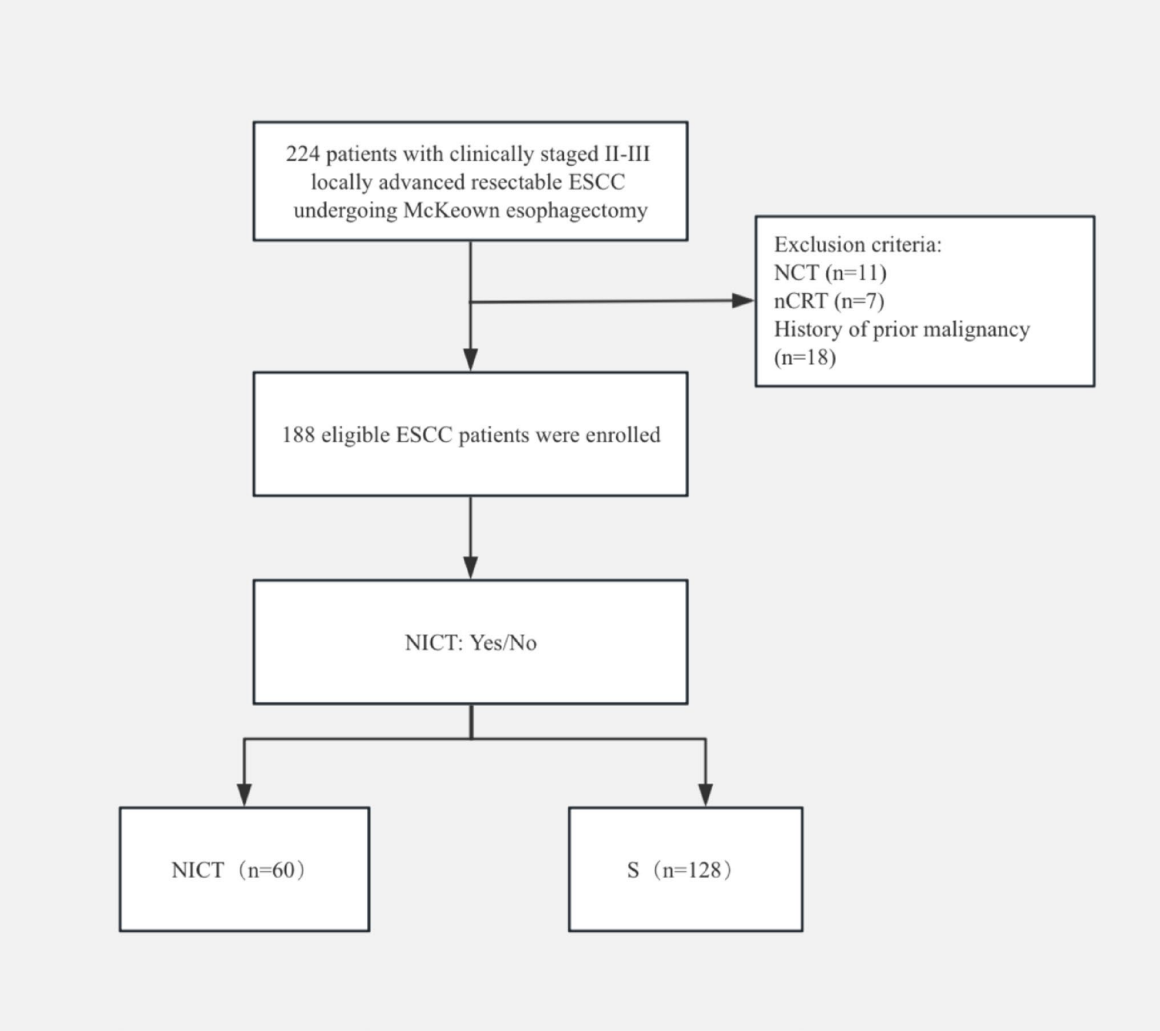
Patient enrollment flowchart. ESCC, esophageal squamous cell carcinoma; NCT, neoadjuvant chemotherapy; nCRT, neoadjuvant chemoradiotherapy; NICT, neoadjuvant immunochemotherapy; S, surgery alone

From December 2019 to May 2023, a total of 224 patients were screened. Exclusions included NCT (n = 11), nCRT (n = 7), and a history of prior malignancy (n = 18). Ultimately, 188 eligible patients were included in this retrospective cohort study, with 60 patients assigned to the NICT group and 128 to the surgery-alone group (Fig. 1). Preliminary comparative analysis showed that baseline clinical characteristics were balanced and comparable between the two groups, with no statistically significant differences observed in any variable (all *P* > 0.05) (Table 1).

**Table 1:** Baseline clinical characteristics

Patient variables	NICT (n = 60)	S (n = 128)	*P*-value
Age, years			0.279
≤ 60	27 (45)	47 (36.7)	
> 60	33 (55)	81 (63.3)	
Gender			0.522
Male	54 (90)	111 (86.7)	
Female	6 (10)	17 (13.3)	
ASA			1.000
I	2 (3.3)	4 (3.1)	
II	57 (95)	121 (94.5)	
III	1 (1.7)	3 (2.3)	
ECOG			0.614
0	40 (66.7)	90 (70.3)	
1	20 (33.3)	38 (29.7)	
Smoking history			0.788
Ever	47 (78.3)	98 (76.6)	
Never	13 (21.7)	30 (23.4)	
Drinking history			0.913
Ever	23 (38.3)	48 (37.5)	
Never	37 (61.7)	80 (62.5)	
BMI (kg/m^2^)	22.2 ± 1.8	22.3 ± 1.7	0.649
Comorbidity			
Hypertension	10 (16.7)	22 (17.1)	0.929
Diabetes mellitus	5 (8.3)	13 (10.2)	0.692
Coronary heart disease	2 (3.3)	2 (1.6)	0.809
Tumor location			0.704
Upper	3 (5)	10 (7.8)	
Middle	41 (68.3)	81 (63.3)	
Lower	16 (26.7)	37 (28.9)	
Clinical TNM Stage			0.185
II	14 (23.3)	42 (32.8)	
III	46 (76.7)	86 (67.2)	
Clinical T stage			0.405
T2	13 (21.7)	35 (27.3)	
T3	47 (78.3)	93 (72.7)	
Clinical N stage			0.721
N0	14 (23.3)	36 (28.1)	
N1	33 (55)	69 (53.9)	
N2	13 (21.7)	23 (18.0)	
Surgical approach			
Robotic-assisted procedure	42 (70)	88 (68.8)	0.863
Thoracoscopic and laparoscopic surgery	18 (30)	40 (31.2)	
Neoadjuvant cycle			
2	46 (76.7)	–	–
> 2	14 (23.3)	–	–
Interval to surgery	36.8 ± 5.5	–	–

Data are presented as n (%) or mean ± standard deviation. Clinical TNM stage, tumor-node-metastasis staging based on clinical and imaging evaluation before treatment

NICT, neoadjuvant immunochemotherapy; S, surgery alone; ECOG, Eastern Cooperative Oncology Group; ASA, American Society of Anesthesiologists

### Perioperative outcomes

Both groups underwent McKeown esophagectomy with standard two-field lymphadenectomy, with no conversion to thoracotomy. The NICT group achieved a significantly higher R0 resection rate [100% (60/60) vs. 91.4% (117/128), *P* = 0.018]. Compared with the surgery-alone group, the NICT cohort had a longer operation time (341.6 ± 51.6 min vs. 302.7 ± 57.7 min, *P* < 0.001). However, no significant differences were observed between groups in intraoperative blood loss, number of lymph nodes dissected, chest tube indwelling time, postoperative ICU readmission, 60-day postoperative mortality, or 30-day readmission (all *P* > 0.05) (Table 2).

**Table 2:** Comparison of perioperative data

	NICT (n = 60)	S (n = 128)	*P*-value
R0 resection	60 (100)	117 (91.4)	0.018
Operation time (min)	341.6 ± 51.6	302.7 ± 57.7	< 0.001
Conversion to thoracotomy	0	0	–
Number of lymph nodes dissected	27.7 ± 9.6	25.0 ± 10.5	0.102
Intraoperative blood loss (ml)	96.6 ± 51.0	97.9 ± 45.4	0.864
Chest tube indwelling time (days)	5.2 ± 1.2	5.3 ± 1.2	0.842
Postoperative ICU readmission	0	2	1.000
Mortality within 60 days postoperatively	0	1	1.000
Readmission within 30 days postoperatively	0	1	1.000

Data are presented as n (%) or mean ± standard deviation

NICT, neoadjuvant immunochemotherapy; S, surgery alone

### Postoperative complications

Postoperative complications were assessed using the Clavien-Dindo classification. The NICT group demonstrated a total complication rate of 40% (24/60), compared with 37.5% (48/128) in the surgery-alone group, with no statistically significant difference. Pulmonary complications were the most frequent postoperative morbidity in both cohorts (35.0% vs. 26.6%, *P* = 0.236), and pneumonia occurred more commonly in the NICT group than in the surgery-alone group (28.3% vs. 21.9%, *P* = 0.333), although the difference was not statistically significant. New-onset atrial fibrillation was the most frequent cardiac complication (3.3% vs. 9.4%, *P* = 0.241). No grade ≥ III complications were observed in the NICT group. In contrast, a total of three patients (2.3%) in the surgery-alone group experienced grade ≥ III complications, including one case (0.8%) of respiratory insufficiency and two cases (1.6%) of gastrointestinal fistula (Table 3).

**Table 3:** Comparison of postoperative complications

	NICT (n = 60)			S (n = 128)			*P*-value		
	Any grade	Grade 1–2	Grade ≥ 3	Any grade	Grade 1–2	Grade ≥ 3	Any grade	Grade 1–2	Grade ≥ 3
Postoperative complications	24 (40)	24 (40)	0	48 (37.5)	48 (37.5)	0	0.742	0.742	–
Pulmonary complications	21 (35)	21 (35)	0	34 (26.6)	34 (26.6)	0	0.236	0.236	–
Pneumonia	17 (28.3)	17 (28.3)	0	28 (21.9)	28 (21.9)	0	0.333	0.333	–
Atelectasis	14 (23.3)	14 (23.3)	0	27 (21.10	27 (21.10	0	0.729	0.729	–
Respiratory insufficiency	3 (5)	3 (5)	0	5 (3.9)	4 (3.1)	1 (0.8)	1.000	0.826	1.000
Pleural effusion	4 (6.7)	4 (6.7)	0	10 (7.8)	10 (7.8)	0	1.000	1.000	–
Gastrointestinal Fistula	7 (11.7)	7 (11.7)	0	18 (14.1)	16 (12.5)	2 (1.6)	0.652	0.871	1.000
Anastomotic stricture	4 (6.7)	4 (6.7)	0	5 (3.9)	5 (3.9)	0	0.646	0.646	–
Poor surgical wound healing	2 (3.3)	2 (3.3)	0	7 (5.5)	7 (5.5)	0	0.785	0.785	–
New-onset atrial fibrillation	2 (3.3)	2 (3.3)	0	12 (9.4)	12 (9.4)	0	0.241	0.241	–
Postoperative Chylothorax	2 (3.3)	2 (3.3)	0	3 (2.3)	3 (2.3)	0	1.000	1.000	–
Postoperative blood transfusion	2 (3.3)	2 (3.3)	0	5 (3.9)	5 (3.9)	0	1.000	1.000	–

Data are presented as n (%)

NICT, neoadjuvant immunochemotherapy; S, surgery alone

### Efficacy evaluation of NICT

The efficacy evaluation outcomes of NICT are summarized in Table 4. Based on the Response Evaluation Criteria in Solid Tumors (RECIST) version 1.1 criteria, radiologic assessment revealed that 8 of 60 patients (13.3%) in the NICT group achieved complete response (CR), while 29 patients (48.3%) exhibited partial response (PR). The objective response rate (ORR), defined as the combined proportion of CR and PR, was 61.7% (37/60). Additionally, 22 patients (36.7%) were categorized as stable disease (SD), and 1 patient (1.7%) progressed to progressive disease (PD). The disease control rate (DCR), calculated as the sum of CR, PR, and SD, reached 98.3% (59/60).

**Table 4:** Efficacy evaluation of NICT

Treatment response evaluation	n (%)
*Radiological assessment*	
CR	8 (13.3)
PR	29 (48.3)
SD	22 (36.7)
PD	1 (1.7)
ORR	37 (61.7)
DCR	59 (98.3)
*Pathological evaluation*	
pCR (TRG1)	17 (28.3)
MPR (TRG1 + TRG2)	37 (61.7)
TRG3/4	23 (38.3)
*ypTNM stage*	
ypT0N0M0 (pCR)	17 (28.3)
I	20 (33.3)
II	10 (16.7)
III	13 (21.7)
*TNM stage downstaging*	
Yes	36 (60)
No	24 (40)
*T stage downstaging*	
Yes	40 (66.7)
No	20 (33.3)
*N stage downstaging*	
Yes	34 (56.7)
No	26 (43.3)

Data are presented as n (%). ypTNM stage, pathological tumor-node-metastasis (TNM) classification assessed after neoadjuvant therapy and surgery

NICT, neoadjuvant immunochemotherapy; CR, complete response; PR, partial response; SD, stable disease; PD, progressive disease; ORR, objective response rate; DCR, disease control rate; pCR, pathological complete response; MPR, major pathological response; TRG, tumor regression grade

All 60 patients in the NICT cohort achieved R0 resection. Postoperative analysis demonstrated a pCR rate of 28.3% (17/60) and an MPR rate of 61.7% (37/60). Comparative evaluation of clinical staging versus post-neoadjuvant pathological staging revealed tumor downstaging in 60% (36/60) of patients, with T-stage downstaging observed in 66.7% (40/60) and N-stage downstaging in 56.7% (34/60) (Table 4).

Patients in the NICT cohort were stratified into three subgroups based on postoperative pathological response: pCR, MPR, and TRG3/4 groups. Statistical analysis demonstrated a significant correlation between histopathological assessment and radiological evaluation following NICT (*P* < 0.001), while no statistically significant association was observed between pathological response and the number of NICT treatment cycles (*P* = 0.813) (Table 5).

**Table 5:** Pathological evaluation of NICT

	Pathological assessment			*P*-value
	pCR (n = 17)	MPR (n = 37)	TRG3/4 (n = 23)	
Imaging assessment				*P* < 0.001
CR	8	8	0	
PR	8	26	3	
SD	1	3	19	
PD	0	0	1	
NICT treatment cycles				*P* = 0.813
2	14	29	17	
>2	3	8	6	

NICT, Neoadjuvant immunochemotherapy; CR, complete response; PR, partial response; SD, stable disease; PD, progressive disease; pCR, pathological complete response; MPR, major pathological response; TRG, tumor regression grade

### Treatment-related adverse events (AEs) of NICT

A comprehensive safety assessment was conducted in 60 patients receiving NICT, incorporating longitudinal follow-up and clinical data analysis per CTCAE v5.0 criteria. AEs of any grade occurred in 96.7% (58/60) of the cohort. Grade 3 AEs were documented in 20% (12/60) of patients, with no grade ≥ 4 events observed. Notably, no treatment discontinuations or mortality attributable to AEs occurred.

The most prevalent grade 3 AEs originated from hematologic systems, ordered by frequency: neutropenia (8.3%, 5/60), leukopenia (6.7%, 4/60), anemia (5.0%, 3/60), and thrombocytopenia (5.0%, 3/60), all of which were managed effectively with supportive care. Non-hematologic AEs predominantly included alopecia (73.3%, 44/60), fatigue (68.3%, 41/60), and nausea (58.3%, 35/60), with only one grade 3 event recorded.

Immune-related adverse events (irAEs) developed in 20% (12/60) of patients, of which 11.7% (7/60) resolved spontaneously within two weeks without medical intervention. Four patients (6.7%) required symptomatic management for irAEs resolution, while one case (1.7%) of immune-mediated pneumonitis necessitated surgical delay (Table 6).

**Table 6:** AEs associated with NICT

	N = 60		
	Any grade AEs	Grade 1 = 2	Grade 3
*Hematological toxicity*			
Leukopenia	22 (36.7)	18 (30.0)	4 (6.7)
Neutropenia	20 (33.3)	15 (25.0)	5 (8.3)
Thrombocytopenia	20 (33.3)	17 (28.3)	3 (5.0)
Anemia	26 (43.3)	23 (38.3)	3 (5.0)
Febrile Neutropenia	0	0	1 (1.7)
*Non-hematological toxicity*			
Nausea	35 (58.3)	35 (58.3)	0
Alopecia	44 (73.3)	44 (73.3)	0
Vomiting	19 (31.7)	18 (30)	1 (1.7)
Dizziness	5 (8.3)	5 (8.3)	0
Diarrhea	14 (23.3)	14 (23.3)	0
Constipation	3 (5.0)	3 (5.0)	0
Fatigue	41 (68.3)	41 (68.3)	0
Arthralgia and bone pain	10 (16.7)	10 (16.7)	0
Myalgia	12 (20.0)	12 (20.0)	0
Rash	8 (13.3)	8 (13.3)	0
Pruritus	12 (20.0)	12 (20.0)	0
*Immunotherapy-related adverse events*			
Elevated Transaminases	5 (8.3)	5 (8.3)	0
Thyroid Dysfunction	8 (13.3)	8 (13.3)	0
Immune-related Pneumonitis	1 (1.7)	0	1 (1.7)

Data are presented as n (%)

NICT, neoadjuvant immunochemotherapy; AEs, adverse events

### Survival outcomes

The median follow-up was 41.4 months [95% Confidence Interval (CI) 40.1–42.7] the entire cohort, with 40.2 months (95% CI 38.8–41.6) in the NICT group and 41.7 months (95% CI 37.6–45.8) in the surgery-alone group. Median DFS and OS were not reached in either group.

**Fig. 2:**
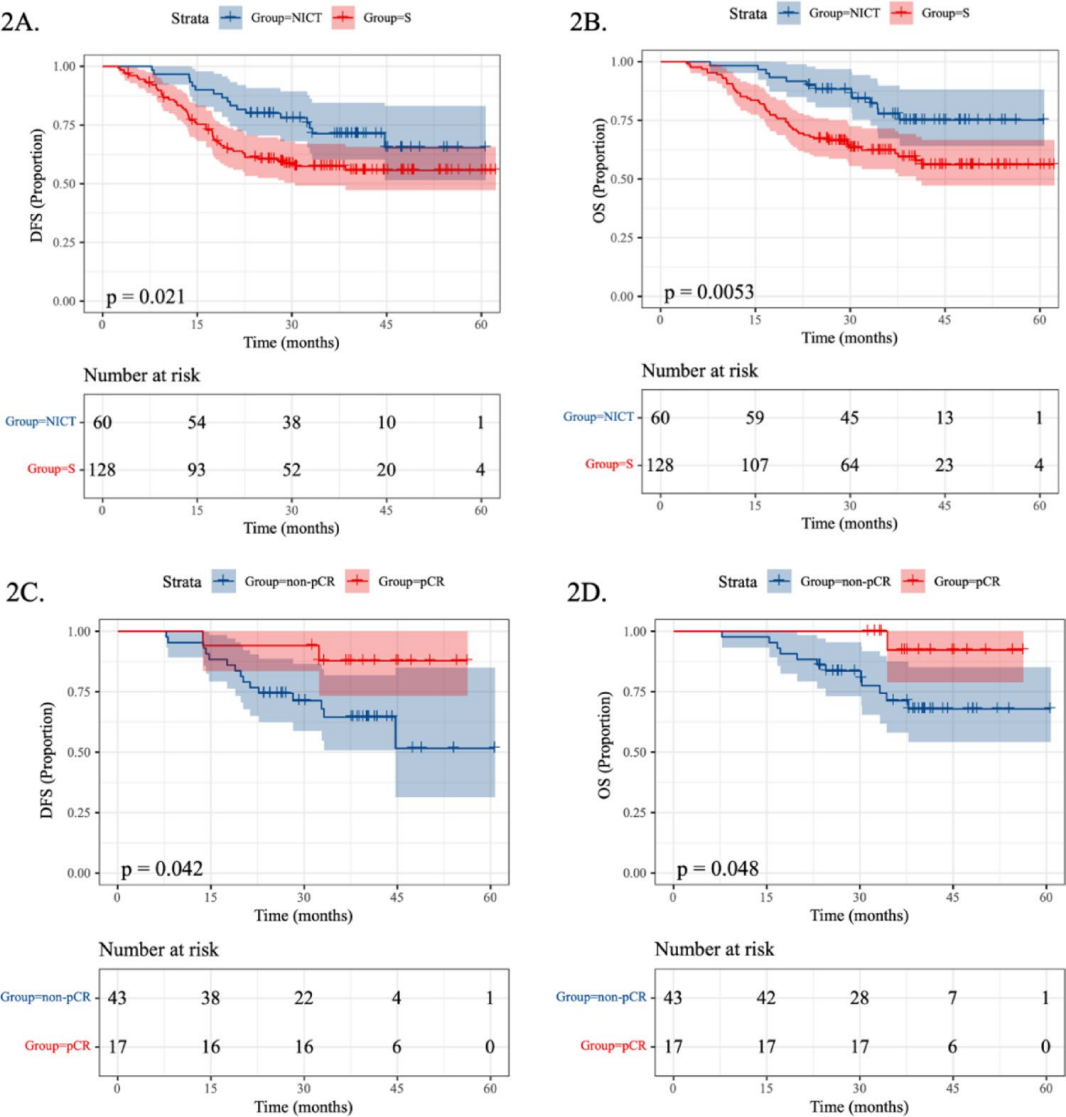

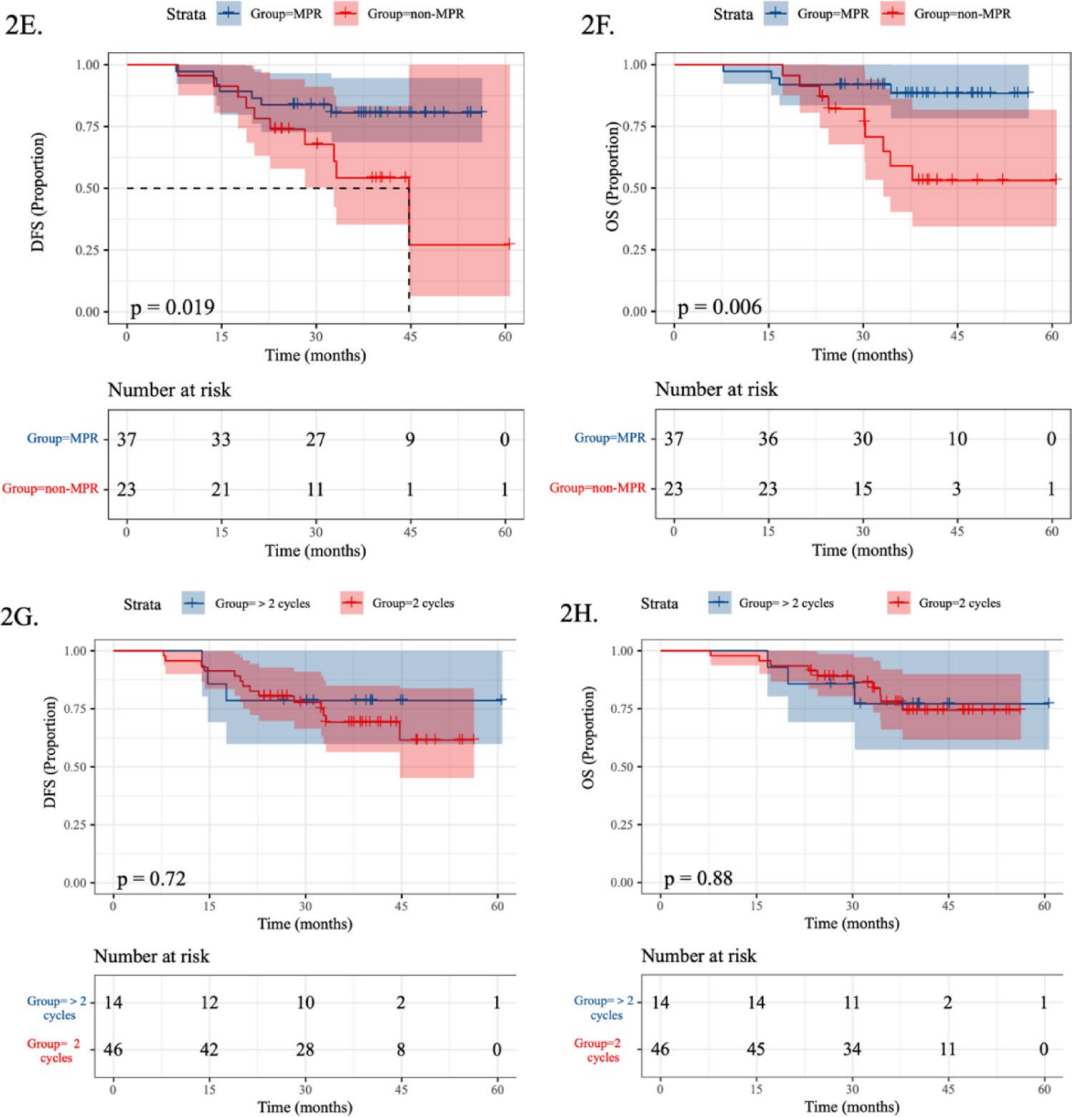
Kaplan–Meier survival analyses comparing outcomes. **A** DFS in NICT vs S groups. **B** OS in NICT vs. S groups. **C** DFS by pCR status in NICT group. **D** OS by pCR status in NICT group. **E** DFS by MPR status in NICT group. **F** OS by MPR status in NICT group. **G** DFS by NICT cycle number (2 vs. > 2). **H** OS by NICT cycle number (2 vs. > 2). NICT, neoadjuvant immunochemotherapy; S, surgery alone, DFS, disease-free survival; OS, overall survival; pCR, pathological complete response; MPR, major pathological response

The NICT group demonstrated superior 1-year DFS (96.7% vs. 81.3%) and OS (98.3% vs. 87.5%) compared to the surgery-alone group. At 2 years, the NICT group also showed higher DFS (76.7% vs. 57.0%) and OS (86.7% vs. 68.0%) rates. NICT was associated with significantly improved DFS (HR 0.547, 95% CI 0.317–0.946; *P* = 0.021) and OS (HR 0.444, 95% CI 0.241–0.816; *P* = 0.0053) (Fig. 2A, B).

The pCR subgroup demonstrated superior 2-year DFS (94.1% vs. 69.8%; HR 0.252, 95% CI 0.057–1.116; *P* = 0.042) and OS (100% vs. 81.4%; HR 0.170, 95% CI 0.022–1.307; *P* = 0.048) compared to the non-pCR subgroup (Fig. 2C, D). Similarly, the MPR subgroup exhibited improved 2-year DFS (91.2% vs. 65.2%; HR 0.359, 95% CI 0.135–0.953; *P* = 0.019) and OS (91.9% vs. 78.3%; HR 0.235, 95% CI 0.072–0.768; *P* = 0.006) versus the non-MPR subgroup (Fig. 2E, F). Patients receiving 2 cycles versus > 2 cycles of NICT showed comparable outcomes in both DFS (76.1% vs. 78.6%; HR 0.713, 95% CI 0.205–2.482; *P* = 0.72) and OS (87.0% vs. 85.7%; HR 0.999, 95% CI 0.275–3.631; *P* = 0.88) (Fig. 2G, H).

Univariate Cox analysis identified ECOG score (HR 5.528, 95% CI 3.308–9.238; *P* < 0.001), coronary heart disease (HR 2.706, 95% CI 0.844–8.670; *P* = 0.096), and treatment approach (HR 0.444, 95% CI 0.241–0.816; *P* = 0.009) as potential OS predictors. Multivariate analysis of factors with *P* < 0.1 confirmed ECOG score (HR 6.778, 95% CI 4.010–11.458; *P* < 0.001), coronary heart disease (HR 4.152, 95% CI 1.244–13.860; *P* = 0.021), and treatment approach (HR 0.285, 95% CI 0.150–0.543; *P* < 0.001) as independent prognostic factors for OS (Table 7).

**Table 7:** Univariate and multivariate cox regression analysis based on baseline characteristics

Characteristics	Number	Univariate analyses		Multivariate analysis	
		HR (95% CI)	*P* value	HR (95% CI)	*P* value
Gender			0.726		
Male	165	0.881 (0.435–1.784)			
Female	23				
Age, years			0.760		
≤ 60	74				
> 60	114	1.081 (0.654–1.787)			
ECOG			< 0.001		< 0.001
0	130				
1	58	5.528 (3.308–9.238)		6.778 (4.010–11.458)	
Smoking history			0.137		
Ever	145	1.637 (0.855–3.136)			
Never	43				
Drinking history			0.711		
Ever	71	1.099 (0.665–1.817)			
Never	117				
Hypertension			0.379		
Yes	32	1.315 (0.715–2.417)			
No	156				
Diabetes mellitus			0.201		
Yes	18	1.583 (0.782–3.205)			
No	170				
Coronary heart disease			0.094		0.021
Yes	4				
No	184	2.706 (0.844–8.670)		4.152 (1.244–13.860)	
Tumor location			0.236		
Upper	13	1.677 (0.709–3.968)			
Middle	122	0.838 (0.486–1.444)			
Lower	53				
Clinical T stage			0.754		
T2	48	1.095 (0.621–1.931)			
T3	140				
Clinical N stage			0.554		
N0-1	152				
N2	36	1.202 (0.653–2.211)			
Surgical approach			0.443		
Robotic-assisted procedure	130				
Thoracoscopic and laparoscopic surgery	58	0.819 (0.491–1.365)			
Treatment approach			0.009		< 0.001
NICT	60	0.444 (0.241–0.816)		0.285 (0.150–0.543)	
S	128				

ECOG, Eastern Cooperative Oncology Group; NICT, neoadjuvant immunochemotherapy; S, surgery alone; HR, hazard ratio; CI, confidence interval

## Discussion

This study analyzed the efficacy and 2-year survival outcomes of NICT in patients with ESCC, and compared them with those of patients who underwent surgery alone during the same period. Our findings suggest that NICT may confer survival benefits over surgery alone without increasing the risk of postoperative complications. It is worth noting that surgery alone was selected as the comparator group to reflect real-world clinical practice in China, where the adoption of neoadjuvant therapy for ESCC remains relatively low. According to a large multicenter retrospective study led by the National Cancer Center of China, only 22.0% of patients received neoadjuvant therapy (Mao et al. 2020). Factors contributing to this include patient and family resistance to preoperative treatment, concerns over potential tumor progression during neoadjuvant therapy, and the increased technical complexity of surgery following radiotherapy. Therefore, the inclusion of a surgery-alone group provides a pragmatic and contextually appropriate comparator for evaluating the real-world efficacy and safety of NICT.

To date, the majority of published studies on NICT have primarily focused on pathological outcomes, with limited survival follow-up data. In our study, 60 patients with locally advanced ESCC received NICT followed by surgery. The pathological pCR rate was 28.3%, MPR rate was 61.7%, while the ORR and disease control rate DCR were 61.7% and 98.3%, respectively. Grade 3 AEs occurred in 20% of patients, with no grade ≥ 4 AEs observed; the incidence of irAEs was 20%. These results are consistent with findings from recent phase II trials. The NICE study, a multicenter, single-arm phase II trial evaluating camrelizumab plus albumin-bound paclitaxel, reported pCR and MPR rates of 39.2% and 68.6% among 51 patients (Yang et al. 2024b). Similarly, the ESCORT-NEO study—the first phase III trial of perioperative immunotherapy in esophageal cancer—reported pCR and MPR rates of 28.0% and 59.1%, respectively, with grade ≥ 3 AEs and irAEs rates of 34.1% and 27.3% (Qin et al. 2024), confirming that NICT provides favorable short-term efficacy and safety in the real-world setting.

As for survival outcomes, the ESCORT-NEO data remain immature, and most published studies reporting survival are single-arm with relatively short follow-up. In our cohort, the 2-year DFS and OS rates in the NICT group were 76.7% and 86.7%, respectively. One study involving 132 ESCC patients who underwent surgery after NICT reported 2-year DFS and OS rates of 55.1% and 78.6% (Guo et al. 2024). Another study reported 2-year DFS and OS rates of 80.7% and 95.7% in the NICT group (Jing et al. 2022). In the NICE-II study, 2-year OS and recurrence-free survival (RFS) rates were 78.1% and 67.9%, respectively (Yang et al. 2024b). The NEOCRTEC5010 trial, a prospective multicenter Randomized Controlled Trial (RCT) based in China evaluating nCRT, reported 2-year DFS and OS rates of 66.4% and 72.7% in the nCRT group, compared with 55% and 63.9% in the surgery-alone group (Yang et al. 2018). These findings suggest that NICT may achieve long-term outcomes comparable to nCRT and has the potential to improve prognosis in patients with locally advanced ESCC.

We also found that patients in the NICT group who achieved pCR or MPR had markedly better survival outcomes. Compared to those who did not achieve pCR, patients in the pCR subgroup demonstrated significantly improved 2-year DFS and OS rates (94.1% vs. 69.8% and 100% vs. 81.4%, respectively). Similarly, the MPR subgroup showed better 2-year DFS and OS rates than the non-MPR subgroup (91.2% vs. 65.2% and 91.9% vs. 78.3%, respectively). This trend has been supported by multiple studies. For example, one study reported 3-year RFS rates of 94.1% vs. 66.3% and 3-year OS rates of 94.1% vs. 72.7% between pCR and non-pCR groups, and 3-year RFS rates of 93.1% vs. 57.9% and OS rates of 97.1% vs. 63.4% between MPR and non-MPR groups (Lv et al. 2024). Another study demonstrated that NICT was associated with better RFS and identified it as an independent prognostic factor (Yang et al. 2023). In the NICE-II study, subgroup analysis showed that 2-year OS and RFS were 91.4% and 77.1% in the MPR group, versus 47.7% and 45.9% in the non-MPR group, respectively, suggesting that MPR is associated with reduced recurrence and improved survival (Yang et al. 2024b).

The optimal duration of NICT remains uncertain. In our study, no statistically significant difference in DFS or OS was observed between patients receiving two cycles versus more than two cycles of NICT, suggesting that prolonged treatment may not yield additional survival benefits. A real-world study reported that the 1-, 2-, and 3-year DFS rates in the two-cycle group were 93.0%, 80.7%, and 77.2%, respectively, while the 3–4-cycle group showed rates of 84.6%, 69.2%, and 65.4%. The corresponding 1-, 2-, and 3-year OS rates were 91.2%, 84.2%, and 80.7% in the 2 cycles group, versus 88.5%, 76.9%, and 71.2% in the 3–4 cycles group (He et al. 2024). Another real-world study comparing 2 versus 4 cycles of neoadjuvant camrelizumab combined with nab-paclitaxel and carboplatin indicated that extended treatment cycles may enhance efficacy without increasing serious adverse events (Zhang et al. 2023). Although these results suggested a survival advantage in the extended-cycle group, evidence also indicates that preoperative neoadjuvant therapy exceeding 2 cycles increases postoperative complications and adverse event incidence, while demonstrating no significant differences in treatment efficacy or prognostic outcomes compared to 2 cycles regimens (Gan et al. 2025; Zhou et al. 2024). Thus, the optimal NICT duration remains to be determined.

This study has several limitations. As a single-center retrospective analysis, it is susceptible to selection bias and limited generalizability. Although multivariate Cox regression was performed to control for confounding variables, unmeasured confounders may still have influenced the survival outcomes. While no statistically significant differences were observed in baseline characteristics between the two groups, treatment decisions in this retrospective cohort were not randomized. All patients were evaluated by a multidisciplinary team and were managed in accordance with national guidelines. For patients eligible for NICT, neoadjuvant therapy was recommended, but some opted for upfront surgery due to personal preferences, concerns about adverse effects, or logistical challenges. This non-random treatment selection may have introduced selection bias and should be considered when interpreting the findings. In addition, the relatively small sample size of the NICT group limited the power of subgroup analyses. Although the median follow-up exceeded 40 months, longer observation is required to assess outcomes such as 5-year survival. Furthermore, the NICT regimens were not fully standardized, which may have introduced treatment heterogeneity.

In summary, our findings suggest that NICT followed by surgery may improve DFS and OS compared to surgery alone in patients with locally advanced ESCC. Moreover, achieving pCR or MPR was associated with better prognosis, indicating that pathological response may serve as a potential surrogate marker for therapeutic benefit. We hope this study contributes meaningful evidence to inform future research and clinical decision-making in this population.

## Conclusions

Compared to surgery alone, NICT followed by surgery was associated with improved 2-year DFS and OS in patients with locally advanced ESCC, without a corresponding increase in postoperative complications. The observed pCR and MPR rates suggest promising short-term pathological efficacy. These findings support the potential role of NICT as a feasible neoadjuvant strategy, though further validation in prospective, large-scale studies is warranted.

## Data Availability

The datasets generated and/or analyzed during the current study are not publicly available due to institutional and patient privacy policies but are available from the corresponding author on reasonable request.
